# Utility of MYD88 in the Differential Diagnosis and Choice of Second-Line Therapy in a Case of Nonsecretory Lymphoplasmacytic Lymphoma versus Free Light Chain Waldenstrom's Macroglobulinemia

**DOI:** 10.1155/2017/5183646

**Published:** 2017-02-12

**Authors:** D. Kazmierski, M. L. Palomba, C. Barsigian

**Affiliations:** ^1^Geisinger Commonwealth School of Medicine, 521 Pine Street, Scranton, PA 18510, USA; ^2^Division of Hematology and Oncology, Memorial Sloan-Kettering Cancer Center, New York, NY, USA; ^3^Hematology & Oncology Associates of Northeastern Pennsylvania, 1100 Meade Street, Dunmore, PA 18512, USA

## Abstract

The MYD88 L265P somatic variant (MYD88) has a high prevalence in Waldenstrom's Macroglobulinemia (WM), a form of lymphoplasmacytic lymphoma (LPL) associated with monoclonal IgM. Although the role of MYD88 in WM was initially reported in 2012, it was not until 2016 that MYD88 testing was included in the National Cancer Care Network (NCCN) Guidelines. We present a case illustrating the utility of MYD88 status in distinguishing atypical forms of WM from marginal zone lymphoma (MZL) and in selecting second-line therapy with ibrutinib. In 2012, a 64-year-old male presented with dyspnea on exertion, a hemoglobin of 5.6 g/dL, a platelet count of 86,000, and monoclonal IgM kappa on serum immunofixation but no detectable M-spike. Bone marrow biopsy revealed 95% monoclonal B-lymphocytes with lymphoplasmacytic differentiation favoring a diagnosis of LPL/WM over MZL, with a favorable response to chemotherapy. This diagnosis was called into question 3 years later following relapse, and MZL was favored based on the lack of MYD88 mutation. One year later, however, repeat bone marrow biopsy detected the MYD88 mutation and therapy with ibrutinib yielded a favorable response. The distinction between certain lymphomas can be problematic and in this case MYD88 was helpful in clarifying a diagnosis of atypical LPL/WM from MZL and in selecting effective second-line therapy.

## 1. Introduction

Waldenstrom Macroglobulinemia (WM), a form of lymphoplasmacytic lymphoma (LPL), is typically defined as being characterized by bone marrow infiltration with monoclonal B-cells in varying stages of maturation (including lymphocytes, lymphoplasmacytic cells, and plasma cells) and the presence of monoclonal IgM on SPEP with the M-spike distinguishing WM from LPL. Nonsecretory LPL constitutes less than 5% of cases of LPL and we are aware of only a few reports of WM focusing on the role of free light chains [1–3], which makes our case unique in that the patient did have elevated free kappa light chains and monoclonal IgM on serum immunofixation but lacked measureable monoclonal IgM on SPEP. Despite these few reports dealing with the role of free light chains in WM, since the initial report of the role of MYD88 in WM in 2012 [4], the literature is rapidly expanding our understanding of the underlying genetic mutations that may play a role in the pathogenesis of LPL/WM. Specifically, the discovery of a highly recurrent somatic mutation (MYD88 L265P) in WM patients is proving useful in the differential diagnosis, prognostic differentiation, and selection of treatment regimens in patients with LPL/WM and related B-cell disorders [4, 5]. In the initial 2012 report conducted by Treon et al. [4], patients with WM underwent whole genome sequencing as well as Sanger Sequencing of bone marrow and paired normal-tissue and results demonstrated a high prevalence of a somatic variant (T→C) that results in an amino acid change (L265P) in MYD88, a gene located at chromosome 3p22.2. The MYD88 L265P variant was absent in bone marrow from healthy donors and absent or rarely expressed in marrow samples from patients with marginal zone lymphoma (MZL), IgM monoclonal gammopathy of undetermined significance (MGUS), and IgM multiple myeloma [4, 5]. Numerous reports have validated these findings [5, 6], revealing a high prevalence of MYD88 L265P among WM patients. For example, Jiménez et al. [6] found the MYD88 L265P variant in 101/117 (86%) of WM patients, 27/31 (87%) of IgM MGUS patients, 9/48 (19%) of nongerminal center DLBCL patients, 3/14 (21%) of MZL patients, and complete absence of the variant in 39 patients with CLL, 35 patients with hairy cell leukemia, 24 patients with multiple myeloma, 25 patients with IgA/IgG MGUS, and 9 patients with LPL. Thus the MYD88 status, when combined with more standard diagnostic tests, may prove useful in differentiating LPL/WM and IgM MGUS from other malignant B-cell neoplasms with similar clinical and pathologic features [7–9]. Despite the expanding knowledge on MYD88 since 2012, it was not until 2016 that testing for MYD88 was added to the essential recommendations for initial work-up of LPL/WM in the NCCN Guidelines. Recent studies have elucidated a mechanistic role for MYD88 L265P in signaling pathways supporting malignant growth involving downstream targets, most notably Bruton Tyrosine Kinase (BTK), which can be exploited with targeted therapy against BTK [7, 8]. In this report, we present a case of a 64-year-old white male who presented in 2012 with clinical features suggestive of nonsecretory LPL or free light chain WM with no monoclonal IgM on SPEP but with a progressive rise in serum free monoclonal kappa light chains, clinical features of WM, and MYD88 L265P detected on progression after first-line therapy.

## 2. Case Presentation

In September 2012, a 64-year-old white male accountant with alcoholism presented from an alcohol rehabilitation facility to a local community hospital ER with dyspnea on exertion and severe anemia with hemoglobin 5.6 g/dL and platelets of 86,000. Serum ferritin was 265 and iron saturation 34% and stools negative for occult blood so gastrointestinal bleed was considered unlikely. The reticulocyte count was very low at 0.86, total bilirubin was 0.9, LDH was 137, haptoglobin was 242, and both the direct and indirect Coombs were negative and thus there was no evidence for hemolytic anemia. There was no quantifiable monoclonal protein on SPEP but a monoclonal IgM kappa was noted on serum immunofixation. Serum viscosity was normal, beta-2 microglobulin was 2.68, and generalized hypogammaglobulinemia was noted with IgG 400 mg/dL, IgA 21 mg/dL, and IgM 53 mg/dL (normal IgM). Serum free light chains and urine electrophoresis were not tested. The AST, ALT, and alkaline phosphatase were normal and hepatitis C and B serologic tests were negative. Given the history of alcohol abuse, abdominal ultrasound and CT scan of abdomen and pelvis were obtained but there was evidence of cirrhosis, hepatosplenomegaly, lymphadenopathy, or ascites.

Peripheral blood smear revealed severe anisocytosis, poikilocytosis, and tear-drop shaped red blood cells and bone marrow biopsy revealed a hypercellular marrow with over 95% diffuse effacement with sheets of lymphocytes and lymphoplasmacytic cells with interspersed plasma cells with scattered Mott cells and Russell bodies. Immunohistochemistry was positive for CD20 and PAX5 but negative for CD10 and BCL-1 and flow cytometry revealed a large monoclonal kappa-restricted B-cell population of 38% that was negative for CD5 and CD10. Karyotype was normal 46XY male and fluorescent in situ hybridization (FISH) revealed 6q- and partial IGH deletion. The final pathology was reported as a B-cell mature neoplasm with plasmacytic differentiation and based on morphology and 6q-; LPL was favored but the differential also included MZL. MYD88 was not tested because it was only initially reported in 2012 [4] and was not part of 2012 NCCN Guidelines (it was not added to NCCN Guidelines until 2016). The patient did not have any symptoms of peripheral neuropathy at diagnosis and so anti-myelin associated glycoprotein antibodies (anti-MAG Ab) were not tested. Based on the marrow findings and an unmeasurable monoclonal IgM by SPEP, an initial diagnosis of LPL was made with the differential also including marginal zone lymphoma.

Because the patient was symptomatic with severe anemia (hemoglobin 5.6 g/dL), he was started on chemotherapy with bortezomib, dexamethasone, and rituximab (BDR) with bortezomib 1.3 mg/m^2^ subcutaneously and dexamethasone 40 mg orally on days 1, 4, 8, and 11, and rituximab 375 mg/m^2^ on day 11 with cycles repeated on a 21-day schedule based on the high response rates reported by Dimopoulos et al. [10] and Treon et al. [11] using twice weekly bortezomib and by Dimopoulos et al. [12] using weekly bortezomib. We chose the twice weekly regimen and since peripheral neuropathy was less severe with subcutaneous bortezomib compared to intravenous in patients with multiple myeloma [13] we administered bortezomib subcutaneously. Treatment with BDR was from 10/2012 through 12/2012 and response was rapid based on improvement of hemoglobin from 5.6 g/dL to 15 g/dL and platelets from 86,000 to 183,000. However, his clinical course deteriorated after cycle 4 with the onset of bilateral hip and leg pain and weakness, severe distal numbness and tingling, and ataxia requiring a walker. MRI of the lumbar spine revealed degenerative changes and foraminal stenosis. Given the absence of monoclonal IgM on SPEP and the normal serum total IgM of 53 mg/dL, rituximab flare (neuropathy due to initial rise in monoclonal IgM) seemed unlikely and the neuropathy was presumptively attributed to the bortezomib, despite the subcutaneous route, and given the disabling symptoms along with a markedly improved hematologic status, treatment was discontinued. Neurology consultation was obtained and nerve conduction studies and peripheral nerve biopsies reveal axonal degeneration and not demyelination and gabapentin helped reduce the “pins and needles” sensation. He remained in apparent remission until 9/2015 (3 years) when hemoglobin dropped from greater than 15 g/dL to 10.2 g/dL and platelets dropped to 70,000 and, for the first and only time, SPEP noted a monoclonal IgM of 0.1 g/dL and serum free kappa light chains were elevated at 122 mg/L.

At that time, he sought a second opinion at Memorial Sloan Kettering and repeat bone marrow biopsy revealed 90% lymphoplasmacytic cells with differential diagnosis based on morphology and IHC of LPL versus MZL. As MYD88 testing had now been added to the 2015 NCCN Guidelines as “useful in certain circumstances” and because he was at a research institution, allele-specific PCR of MYD88 was performed on the bone marrow but was not detected. Based partially on the negative MYD88, a diagnosis of MZL was considered more likely than LPL/WM and because he was asymptomatic with stable hemoglobin and platelets, observation was chosen. After 8 months of observation, hemoglobin dropped further to 8 g/dL and serum free kappa light rose to 356 mg/L and repeat bone marrow biopsy 6/2016 revealed >95% lymphoplasmacytic cell infiltrate and, this time, allele-specific PCR was positive for MYD88 L265P and next generation sequencing revealed wild-type CXCR4. Based on the newly detected MYD88 L265P mutation and wild-type CXCR4, ibrutinib 420 mg orally daily was started and after 1 month hemoglobin increased to >10 g/dL and platelets to >100,000 and after 5 months of ibrutinib he continues to improve with hemoglobin 10.7 g/dL and platelets 147,000 and drop of free kappa light chains from 356 mg/L to 58 mg/L and kappa : lambda ratio from 27 to 5. Therapy was tolerated well except for nausea and vomiting requiring dosage adjustment from 420 mg daily to 240 mg. Neuropathy is improving and anti-myelin associated glycoprotein antibodies were tested for the first time 8/12/2016 but were not detected.

## 3. Discussion

Our case highlights salient features of classical LPL/WM and also presents some of the complexities encountered in the differential diagnosis of this disorder from other B-cell neoplasms. We also focus on new developments regarding the MYD88 status (added to NCCN Guidelines for the first time in 2016) in both the differential diagnosis of LPL/WM from other B-cell neoplasms (in this case, LPL/WM versus MZL) as well as in the selection of potential targeted therapies. A unique aspect of this case, in our opinion, was the absence of measurable monoclonal IgM on SPEP but a detectable monoclonal IgM band on serum immunofixation along with elevated serum free kappa light chains (currently not a component of NCCN Guidelines for diagnosis or management of LPL/WM). Although the World Health Organization and the WM International Workshop (WMIWS) criteria for WM state that IgM monoclonal gammopathy of any concentration distinguishes WM from LPL, the unmeasurable amounts of monoclonal IgM in our patient made the distinction of WM from LPL and MZL perplexing. It was only after relapse that we started to measure serum free lights (as is routinely done in multiple myeloma) and found that the patient did indeed have very elevated free kappa light chains that were monoclonal based on high kappa : lambda ratio of prior to initiation of second-line therapy with ibrutinib. The effectiveness of ibrutinib was assessed by following free kappa light chain levels and kappa : lambda ratios as opposed to IgM levels (as recommended by NCCN Guidelines and WMIWS criteria).

In reviewing the literature, we found only a few reports on the role of free light chains in WM [1–3] and it seems that free light chains are more sensitive than monoclonal IgM in assessing response to therapy and/or progression of disease [1] and that prognosis may be correlated with free light chain levels with 60 mg/L favoring good prognosis and >60 mg/L portending a poor prognosis [2]. However, we are unaware of any cases of WM characterized only by elevated free light chains without measurable monoclonal IgM and our case raises the possibility that WM may include patients with free light only disease as can be seen for multiple myeloma. It is also possible that our patient had cytoplasmic IgM (since Mott cells and Russell bodies were noted on the bone marrow) but failed to secrete monoclonal IgM as reported for a case of IgM multiple myeloma [3]. Finally, although it is possible that monoclonal IgM was undetectable in the serum due to the presence of cold agglutinins or cryoglobulins, however hemolytic anemia was absent and cold agglutinins were undetected. We did not initially screen for cryoglobulins as we felt cryoglobulinemia was unlikely since the patient tested negative for hepatitis C. At second opinion at Memorial Sloan Kettering, however, cryoglobulins were detected. Thus it is theoretically possible that had SPEP and serum IgM levels been determined on specimens maintained under warm bath conditions, monoclonal IgM may have been measurable on SPEP.

In our patient, the presence of MYD88 at bone marrow biopsy on relapse in 2016 along with increased monoclonal free kappa light chains contributes evidence (albeit not exclusive) favoring a diagnosis of WM over LPL since MYD88 is now known to be present in 95% of WM cases [8, 9] but was reported to be absent in 9 cases of LPL [6]. In a similar way, since MYD88 has not been detected in multiple myeloma [6], a recent report used MYD88 negativity to help establish a diagnosis of nonsecretory IgM multiple myeloma from WM in a patient that secreted only free light chains but who had cytoplasmic IgM [3]. In reviewing our case, a question that arises is why he did not initially test positive for the MYD88 mutation at the bone marrow biopsy done at second opinion in 2015 but did with the third bone marrow when relapse was clinically evident in 2016. Although it is clear that the methods used to detect the mutation are important and that the sensitivities of these methods vary as evidenced by the fact that the initial report of Treon et al. [4] detected the mutation in 70% of LPL/WM patients by whole genome sequencing performed on magnetic bead-isolated CD19+ mononuclear B-cells but the mutation has since been detected in 93 to 97% of these patients by allele-specific PCR [8], the negative MYD88 at second opinion was by allele-specific PCR but may simply have been a false negative. We do not feel that there was transformation of the patient's lymphoma at any point since marrow morphology, immunohistochemistry, and flow cytometry were essentially the same on three successive marrows. The finding of MYD88 on the third marrow, despite the previous negative result, underlines the importance of serial testing when the clinical picture supports a diagnosis of WM and particularly when the choice of second-line therapy would be influenced by the results.

In this regard, the MYD88 status is now known to influence the overall prognosis as well as response to targeted therapies such as ibrutinib in patients with LPL/WM [7, 8]. Specifically, the efficacy of ibrutinib in previously treated LPL/WM patients revealed “major responses” with response rates highest among patients with MYD88 L265P mutation and wild-type CXCR4 suggesting [7]. Based on the MYD88 L265P mutation and wild-type CXCR4 on relapse, our patient was started on ibrutinib and continues to improve in all respects.

## 4. Conclusion

This case highlights salient features of classical WM, describes difficulties that can be encountered in establishing a diagnosis of WM when monoclonal IgM and total serum IgM levels are extremely low, expands upon the concept of “free light chain only” WM, comments on the recent addition of MYD88 L265P testing to the 2016 NCCN Guidelines, reiterates the role of MYD88 in both the differential diagnosis of WM from other forms of NHL, and briefly highlights the influence of the MYD88 status on response to targeted therapy with the Bruton Tyrosine Kinase inhibitor, ibrutinib.
